# Measurement of Heart Rate Using the Polar OH1 and Fitbit Charge 3 Wearable Devices in Healthy Adults During Light, Moderate, Vigorous, and Sprint-Based Exercise: Validation Study

**DOI:** 10.2196/25313

**Published:** 2021-03-25

**Authors:** David Joseph Muggeridge, Kirsty Hickson, Aimie Victoria Davies, Oonagh M Giggins, Ian L Megson, Trish Gorely, Daniel R Crabtree

**Affiliations:** 1 Edinburgh Napier University Edinburgh United Kingdom; 2 Division of Biomedical Sciences University of the Highlands and Islands Inverness United Kingdom; 3 Department of Nursing and Midwifery University of the Highlands and Islands Inverness United Kingdom; 4 NetwellCASALA Dundalk Institute of Technology Dundalk Ireland

**Keywords:** heart rate, photoplethysmography, wearable electronic devices, validation study, exercise, mobile phone

## Abstract

**Background:**

Accurate, continuous heart rate measurements are important for health assessment, physical activity, and sporting performance, and the integration of heart rate measurements into wearable devices has extended its accessibility. Although the use of photoplethysmography technology is not new, the available data relating to the validity of measurement are limited, and the range of activities being performed is often restricted to one exercise domain and/or limited intensities.

**Objective:**

The primary objective of this study was to assess the validity of the Polar OH1 and Fitbit Charge 3 devices for measuring heart rate during rest, light, moderate, vigorous, and sprint-type exercise.

**Methods:**

A total of 20 healthy adults (9 female; height: mean 1.73 [SD 0.1] m; body mass: mean 71.6 [SD 11.0] kg; and age: mean 40 [SD 10] years) volunteered and provided written informed consent to participate in the study consisting of 2 trials. Trial 1 was split into 3 components: 15-minute sedentary activities, 10-minute cycling on a bicycle ergometer, and incremental exercise test to exhaustion on a motorized treadmill (18-42 minutes). Trial 2 was split into 2 components: 4 × 15-second maximal sprints on a cycle ergometer and 4 × 30- to 50-m sprints on a nonmotorized resistance treadmill. Data from the 3 devices were time-aligned, and the validity of Polar OH1 and Fitbit Charge 3 was assessed against Polar H10 (criterion device). Validity was evaluated using the Bland and Altman analysis, Pearson moment correlation coefficient, and mean absolute percentage error.

**Results:**

Overall, there was a very good correlation between the Polar OH1 and Polar H10 devices (*r*=0.95), with a mean bias of −1 beats·min^-1^ and limits of agreement of −20 to 19 beats·min^-1^. The Fitbit Charge 3 device underestimated heart rate by 7 beats·min^-1^ compared with Polar H10, with a limit of agreement of −46 to 33 beats·min^-1^ and poor correlation (*r*=0.8). The mean absolute percentage error for both devices was deemed acceptable (<5%). Polar OH1 performed well across each phase of trial 1; however, validity was worse for trial 2 activities. Fitbit Charge 3 performed well only during rest and nonsprint-based treadmill activities.

**Conclusions:**

Compared with our criterion device, Polar OH1 was accurate at assessing heart rate, but the accuracy of Fitbit Charge 3 was generally poor. Polar OH1 performed worse during trial 2 compared with the activities in trial 1, and the validity of the Fitbit Charge 3 device was particularly poor during our cycling exercises.

## Introduction

### Background

Consumer wearables constitute an ever-evolving industry with applications across multiple sectors of society. One key demand for wearable technology is to monitor and use parameters associated with physical activity for sport performance, health, and well-being. For example, a recent systematic review and meta-analysis concluded that the utilization of a consumer-based wearable activity tracker, used either as the primary component of an intervention or as part of a broader physical activity intervention, has the potential to increase participation in physical activities [1].

The autonomic nervous system (ANS) is interlinked with many physiological systems, and heart rate (HR) measures are considered surrogate markers of ANS status [2,3]. Exercise training stimulates ANS status changes, and HR measures therefore reflect physiological responses related to training adaptations [4]. HR monitoring enables noninvasive, cost-effective, and continuous insights into exercise intensity [5]. This may be important for optimizing training responses and for safety considerations (eg, restricting exercise intensity because of contraindications associated with an elevated HR). Wrist-worn, optical HR sensors using photoplethysmography (PPG) technology are exciting, as they reduce the requirement for chest strap or electrocardiogram (ECG) device use. ECG devices are inconvenient, can cause discomfort, and may be time-consuming to use, potentially providing a barrier to participation and optimal training. Although the use of PPG technology is not new in devices, the range of activity types and intensities being performed in validation studies is often restricted. Despite the widely adopted use of PPG in wearable devices, the standards of accuracy and reliability outside of medical devices can be poor, technology frequently advances, and new products are continuously released. Furthermore, the algorithms adopted by companies to derive HR from raw PPG signals are constantly changing, thereby affecting the validity of wearable devices for measuring HR. It is therefore important that devices are continually subjected to scientific scrutiny for appropriate interpretation and advice to be provided.

Polar OH1 (Polar Electro Oy) and Fitbit Charge 3 (Fitbit Inc) are two of the latest available devices that use PPG technology. They differ in that the Fitbit device constitutes a watch worn on the wrist, whereas Polar OH1 may be worn on either the forearm or the temple and is a stand-alone optical HR sensor. It has previously been shown that Polar OH1 is accurate at measuring HR during moderate-intensity yoga compared with a Polar H7 chest strap (mean bias: −0.76 beats·min^-1^; 95% limits of agreement [LoAs]: −3.83 to 5.35 beats·min^-1^) [6] and during moderate-to-high intensity treadmill and spin bike exercise compared with ECG (forearm sensor aggregated data mean bias: 0.27 beats·min^-1^; LoAs: −4.68 to 5.22 beats·min^-1^) [7]. Conversely, although the validity of Fitbit Charge 2 has previously been assessed [8,9], we are unaware of any study assessing the validity of Fitbit Charge 3. Fitbit Charge 3 was released in 2018 as an upgrade to the subsequently discontinued Fitbit Charge 2; however, despite Fitbit Charge 2 demonstrating poor HR measurement accuracy at higher workloads [8,10] and during cycling [9,11], HR monitoring technology appears unchanged. Furthermore, limited data are available for a range of exercise types and intensities. Specifically, there are currently no studies that have assessed PPG HR validity during sprint interval exercise (SIE), defined as exercise bouts performed in an *all-out* manner or at an absolute intensity that exceeds the workload required to elicit maximal oxygen uptake, with each bout separated by a recovery interval [12]. This may be particularly pertinent given the current popularity of SIE and high-intensity interval exercise (HIIE), which is commonly defined as relatively intense bouts of exercise that elicit ≥80% of maximal HR, interspersed with recovery periods [12]. Several studies examining the validity of commercial wearable devices have reported an increase in HR measurement error as exercise intensity increases [8,9,13,14]. This has been attributed to increased motion artifacts caused by rapid arm swinging during running [13], although it has been suggested that sustained isometric muscle contractions when gripping handlebars during cycling may reduce contact between a wrist-worn device and the skin [15]. In addition, during the initiation of exercise, HR can change rapidly with a limited increase in forearm or wrist blood flow to the skin [16]. Therefore, it is important to assess the validity of HR measurements during different activities and intensities. To our knowledge, no study has investigated the accuracy of Polar OH1 or Fitbit Charge 3 across a range of activity types and intensities.

### Objectives

Therefore, the objective of this study was to assess the validity of Polar OH1 and Fitbit Charge 3 for measuring HR during rest, cycling, walking, and running activities. Validity during cycling, walking, and running activities was assessed across a range of intensities, including light, moderate, vigorous, and uniquely sprint-type exercise.

## Methods

### Participants

A total of 20 healthy adults (9 female; height: mean 1.73 [SD 0.1] m; body mass: mean 71.6 [SD 11.0] kg; fat mass: mean 17.0% [SD 7.8%]; age: mean 40 [SD 10] years; and International Physical Activity Questionnaire-Short Form [IPAQ-SF] [17] Physical Activity Category: high=20, moderate=0, and low=0) volunteered and provided written informed consent to participate in the study. Inclusion criteria were age between 18 and 60 years and categorized as having moderate-to-high levels of physical activity (as determined by the IPAQ-SF). Exclusion criteria were categorized as having low levels of physical activity (as determined by the IPAQ-SF), a noncommunicable disease (eg, cardiovascular disease, cancer, and respiratory disease), musculoskeletal injury in the past 2 months, and illness in the previous 6 weeks. One female participant withdrew from the study at visit 1 because of injury (n=19). All the remaining participants completed visit 1 (n=19); however, 1 female participant was unable to complete visit 2 because of illness (n=18), and 1 male participant was unable to complete the visit-2 treadmill sprints because of injury (treadmill sprints; n=17). Ethical approval was provided by the University Ethics Committee at the University of the Highlands and Islands (OL-ETH-SHE-1436). All procedures were conducted in accordance with the Declaration of Helsinki 1974 and its later amendments. Written informed consent was obtained from all volunteers before entering the study, and the participants could withdraw at any point.

### Study Design

The study consisted of 2 visits to the Active Health Exercise Laboratory at the University of the Highlands and Islands, Inverness, which were conducted a minimum of 3 days apart. Participants were asked to refrain from intense physical activity (24 h), alcohol (12 h), caffeine (6 h), and food (3 h) before arrival at the laboratory for each visit. During visit 1, participants completed 15 minutes of sedentary activities, 10 minutes of cycling, and a treadmill protocol. For visit 2, each participant completed cycling and treadmill-based HIIE protocols. During each of the trials, participants’ HRs were continuously monitored by a Polar H10 heart rate monitor (Polar Electro Oy; criterion measure), Polar OH1, and Fitbit Charge 3.

### Devices

#### Polar H10 HR Monitor

Polar H10 was used as the criterion device. The HR sensor was attached to a Polar Pro heart rate strap placed over the sternum. Polar H10 live data were transmitted to a spiroergometry system (METALYZER 3B, CORTEX Biophysik GmbH), which recorded HR data at 1-second intervals. Polar H10 has previously been found to be valid when compared with ECG, with a correlation of *r*=0.997 [18].

#### Polar OH1

Polar OH1 was attached to an arm band and strapped securely to the nondominant forearm, according to the manufacturer’s instructions. HR data were recorded at 1-second intervals using 6 light-emitting diode sensors and live transmitted via Bluetooth to a smartphone with the Polar Beat app (Polar Electro Oy). After completion of each visit, data were uploaded to the Polar Flow web service (Polar Electro Oy).

#### Fitbit Charge 3

Fitbit Charge 3 was attached to the nondominant wrist, 2-finger widths above the ulnar styloid process, following the manufacturer’s instructions. According to the manufacturer, Fitbit Charge 3 uses *PurePulse* wrist HR technology to measure HR. Data were synced to an anonymized Fitbit account, and subsequently, the intraday second-by-second data were exported for each session using the opensource software *Pulse Watch* [19].

### Study Procedures

#### Visit 1

Upon arrival at the laboratory, participants were briefed on the protocol before anthropometric variables were measured. Height, body mass, and body composition were measured with participants wearing light exercise clothing. Height was measured to the nearest 0.1 cm using a portable stadiometer (Model 213, Seca), body mass was measured to the nearest 0.1 kg using a floor scale (Model 875, Seca), and body composition was assessed using bioelectrical impedance (MC780MA, Tanita Corporation). The HR measuring devices were subsequently attached as described above.

The trial was split into 3 components. Component 1 consisted of 15 minutes of sedentary activities. The participants remained seated in a chair for the duration of component 1 and were instructed to keep their movement to a minimum. During the first 5 minutes, participants sat quietly before watching 5 minutes of a nature documentary. All participants watched the same 5-minute section of the documentary. Finally, the participants completed a cognitive task where they were provided with a choice of either a word search, crossword, or sudoku puzzle and were instructed to complete as much as possible within the time frame. Before component 2, a target HR range was calculated to determine 60%-85% of HR reserve target intensity using the equation:


Target HR = Percentage target intensity × (age-predicted max HR − resting HR) + resting HR

Resting HR was calculated as an average of the final minute of sitting quietly, whereas the age-predicted maximum HR was calculated using the following formula by Gellish et al [20]:

Age-predicted max HR = 206.7 − (0.67 × age).

Component 2 consisted of 10-minute cycling on a bicycle ergometer (Lode Corival). During the cycling tasks, each participant completed 5 minutes of light work at 50 W. After 5 minutes, the intensity was increased to substantially elevate HR to between 60% and 85% of the HR reserve. Between components 2 and 3, participants rested for a minimum of 10 minutes.

Component 3 consisted of an incremental exercise test on a motorized treadmill (Skillrun, Technogym). The treadmill test consisted of a range of intensities from light to high intensity, increasing at 3-minute intervals until volitional exhaustion. The initial phase of each test was continuous, beginning with walking at speeds of 4, 5, and 6 km·h^-1^ and then running at speeds of 8 and 10 km·h^-1^ at 1% gradient for 3 minutes. For those who were able to continue, a subsequent discontinuous phase of the test immediately followed the continuous phase. The discontinuous phase consisted of running for 3 minutes at 12, 14, 16, and 18 km·h^-1^ (or until volitional exhaustion), with each stage separated by 3 minutes of active recovery (walking at 4 km·h^-1^). The discontinuous phase was used to allow the participants to complete as many stages as possible. Participants were not required to complete all stages to be included in the analysis.

#### Visit 2

Upon arrival at visit 2, the devices were attached to the participants, as described in visit 1. Visit 2 was split into 2 components. Component 1 consisted of a 3-minute warm-up followed by 4 maximal sprints, each lasting 15 seconds and interspersed with 3-minute active recovery on a cycle ergometer (Wattbike Pro, Wattbike). The airbrake resistance was set at 1 during the warm-up and active recovery phases and was increased based on body mass, as per the manufacturer’s guidelines (Multimedia Appendix 1), immediately before each sprint. Following the completion of a 3-minute active cooldown, participants were instructed to rest for 30 minutes before commencing component 2.

Component 2 was performed using the Technogym Skillrun treadmill’s *parachute training mode*, which is a nonmotorized program and whose objectives are based on distance, not duration. In addition, treadmill belt resistance is adjusted by changing the *parachute size*, which ranges from extra small (lowest resistance setting) to extra large (highest resistance setting); however, the resistance that each parachute size pertains to has not been quantified by Technogym. For the purposes of this study, the distance to be covered was selected based on the final velocity of the incremental treadmill test at visit 1 (Multimedia Appendix 2), and the treadmill belt resistance was set based on body mass (Multimedia Appendix 3). Participants self-propelled the treadmill belt and were secured to the treadmill using a harness worn around their waist. Before starting the repeated sprint protocol, participants completed 400 m on the treadmill at a self-selected pace, with belt resistance at the lowest setting, to act as a familiarization and warm-up. They were then given the opportunity to perform the stretching exercises. The sprints consisted of 4 repetitions, each lasting approximately 15 seconds (range 13-18 s). It was anticipated that the duration of each sprint would last approximately 15 seconds to match the sprint cycling trial, as it is not possible to complete a parachute test on the treadmill in a time setting. Following each sprint, the treadmill was set to a motorized setting of 4 km·h^-1^ and 1% gradient for 3 minutes of active recovery.

### Data Analysis

Data from the 3 devices were time-aligned and split into the following parts:

- Visit 1: rest, light cycling, vigorous cycling, and treadmill
- Visit 2: sprint cycling and sprint running

The validity of Polar OH1 and Fitbit Charge 3 was compared with the validity of Polar H10 (criterion device) for all data points and as an average HR for each segment. Data alignment and filtering were performed in R Studio using the packages dplyr and tidr. Before analysis, the normality of data was assessed using histograms and quantile-quantile plots. Validity was subsequently evaluated using the Bland and Altman [21] analysis. Secondary measures, including the Pearson moment correlation coefficient and mean absolute percentage error (MAPE), are also provided. The Bland and Altman [21] analysis was used to express agreement between the measured and predicted beats per minute, where 95% LoA was calculated as mean bias (1.96SD). We deemed a MAPE of 0%-5% to be within the acceptable limits, a commonly adopted approach [9,22]. Correlation coefficients were interpreted as very poor (*r*<0.69), poor (*r*=0.70-0.84), good (*r*=0.85-0.94), very good (*r*=0.95-0.994), and excellent (*r*>0.995). The MAPE was calculated using the following equation, which provided a general measurement error for the monitors:

MAPE=((monitor−criterion)/criterion100)

## Results

### Validity of HR Across All Data

Combined data across all activity types showed that Polar OH1 underestimated HR by 1 beat·min^-1^ (LoA: −20 to 19 beats·min^-1^) versus the Polar H10 device, and there was a very good correlation between the devices (*r*=0.957; Table 1). The Fitbit device underestimated the HR by 7 beats·min^-1^ (LoA: −46 to 33 beats·min^-1^; Table 1). Overall, there was a good correlation between Fitbit Charge 3 and Polar H10 (*r*=0.807), and the MAPE for both devices was deemed acceptable (Table 1). In addition, aggregated HR data for each activity domain revealed results similar to those of the unaveraged data analysis (Table 2).

**Table 1:** Validity of measuring heart rate with the Polar OH1 and Fitbit Charge 3 devices. Data are unaveraged across all data points (overall) and within each of the activity domains.

Device		Overall (n=35,639^a^)	Rest (n=5448^a^)	Cycling light (n=1903^a^)	Cycling hard (n=1928^a^)	Treadmill (n=14,489^a^)	Sprint cycling (n=5516^a^)	Sprint running (n=6355^a^)
**Polar H10**								
	Heart rate (beats·min^-1^), mean (SD)	114 (33)	63 (10)	89 (12)	119 (16)	124 (31)	131 (25)	123 (19)
**Polar OH1**								
	Heart rate (beats·min^-1^), mean (SD)	113 (33)	63 (10)	89 (12)	118 (17)	124 (30)	125 (26)	125 (20)
	Mean bias (beats·min^-1^)	−1	0	1	−1	0	−6	2
	Limits of agreement (beats·min^-1^)	−20 to 19	−4 to 4	−5 to 7	−7 to 5	−9 to 8	−38 to 27	−27 to 31
	MAPE^b^ (%)	0.4	0.2	−0.8	0.6	0.2	3.9	−1.9
	Correlation coefficient	0.957	0.974	0.983	0.985	0.990	0.794	0.722
	95% CI of correlation coefficient	0.956 to 0.958	0.973 to 0.975	0.981 to 0.984	0.983 to 0.987	0.990 to 0.991	0.784 to 0.803	0.710 to 0.734
**Fitbit Charge 3**								
	Heart rate (beats·min^-1^), mean (SD)	107 (31)	62 (9)	81 (12)	98 (24)	123 (27)	111 (30)	114 (16)
	Mean bias (beats·min^-1^)	−7	−1	−7	−21	−1	−20	−10
	Limits of agreement (beats·min^-1^)	−46 to 33	−7 to 5	−38 to 23	−70 to 29	−30 to 28	−79 to 40	−49 to 30
	MAPE (%)	−4.4	−1.4	−7.1	−16.4	0.7	−13.5	−6.3
	Correlation coefficient	0.807	0.946	0.272	0.183	0.879	0.390	0.348
	95% CI of correlation coefficient	0.804 to 0.811	0.943 to 0.949	0.230 to 0.328	0.127 to 0.238	0.875 to 0.882	0.368 to 0.412	0.326 to 0.369

^a^n: number of data points analyzed for each domain.

^b^MAPE: mean absolute percentage error.

**Table 2:** Validity of measuring heart rate with Polar OH1 and Fitbit Charge 3 devices. Data are aggregated to a single data point for each of the activity domains. Data are analyzed for all data points (column “Overall”) and for each of the activity domains.

Device			Overall (n=111^a^)		Rest (n=19^a^)		Cycling light (n=19^a^)		Cycling hard (n=19^a^)		Treadmill (n=19^a^)		Sprint cycling (n=18^a^)		Sprint running (n=17^a^)
**Polar H10**															
	Heart rate (beats·min^-1^), mean (SD)	106 (27)		62 (9)		89 (11)		119 (12)		124 (14)		128 (17)		120 (12)	
**Polar OH1**															
	Heart rate (beats·min^-1^), mean (SD)	105 (27)		62 (9)		89 (10)		118 (12)		124 (14)		122 (17)		121 (15)	
	Mean bias (beats·min^-1^)	1		0		0		1		0		5		1	
	Limits of agreement (beats·min^-1^)	−8 to 10		0 to 1		−3 to 2		−2 to 4		−1 to 2		−8 to 18		−16 to 15	
	MAPE^b^ (%)	0.6		0.2		0.7		−0.8		0.2		−4.1		0.5	
	Correlation coefficient	0.954		0.983		0.974		0.985		0.992		0.807		0.67	
	95% CI of correlation coefficient	0.953 to 0.955		0.982 to 0.984		0.971 to 0.977		0.083 to 0.987		0.992 to 0.993		0.795 to 0.819		0.651 to 0.687	
**Fitbit Charge 3**															
	Heart rate (beats·min^-1^), mean (SD)	97 (26)		61 (9)		81 (10)		100 (21)		123 (10)		109 (23)		112 (11)	
	Mean bias (beats·min^-1^)	−9		−1		−7		−19		−2		−18		−8	
	Limits of agreement (beats·min^-1^)	−41 to 23		−2 to 0		−36 to 22		−63 to 26		−13 to 10		−63 to 26		−28 to 12	
	MAPE (%)	7.37		−1.5		−7		−15		−1		−13.9		−6	
	Correlation coefficient	0.888		0.884		−0.056		0.183		0.924		0.771		0.496	
	95% CI of correlation coefficient	0.885 to 0.890		0.876 to 0.891		−0.113 to 0.002		0.127 to 0.238		0.921 to 0.927		0.756 to 0.784		0.471 to 0.520	

^a^n: number of data points analyzed for each domain.

^b^MAPE: mean absolute percentage error.

### Validity of HR for Each Activity Type

**Figure 1:**
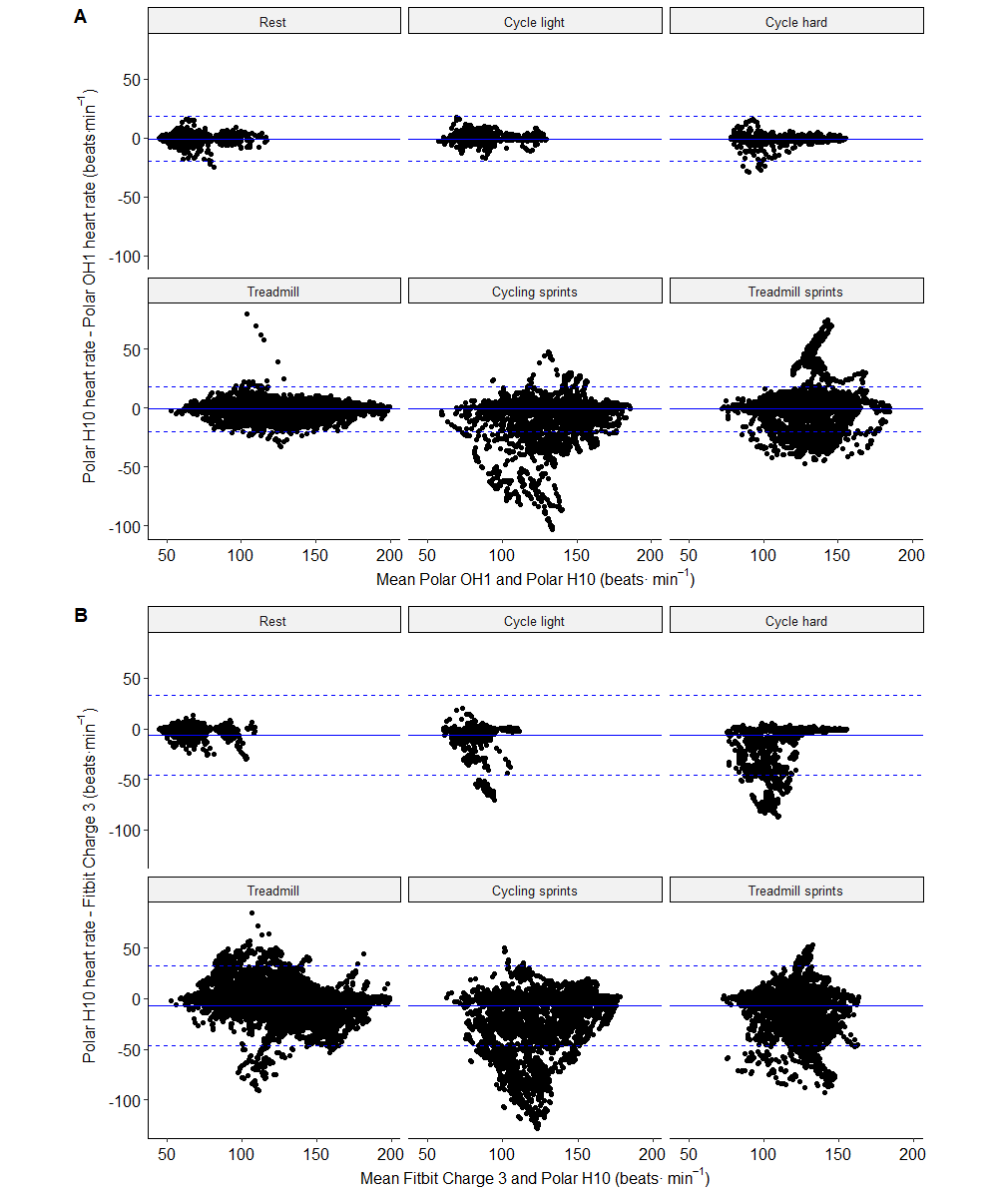
Bland and Altman plots for unaveraged data across each activity domain. Subpart A shows data from Polar OH1, and subpart B shows data from Fitbit Charge 3. Solid blue line represents the mean bias, and blue dashed lines represent the limits of agreement.

**Figure 2:**
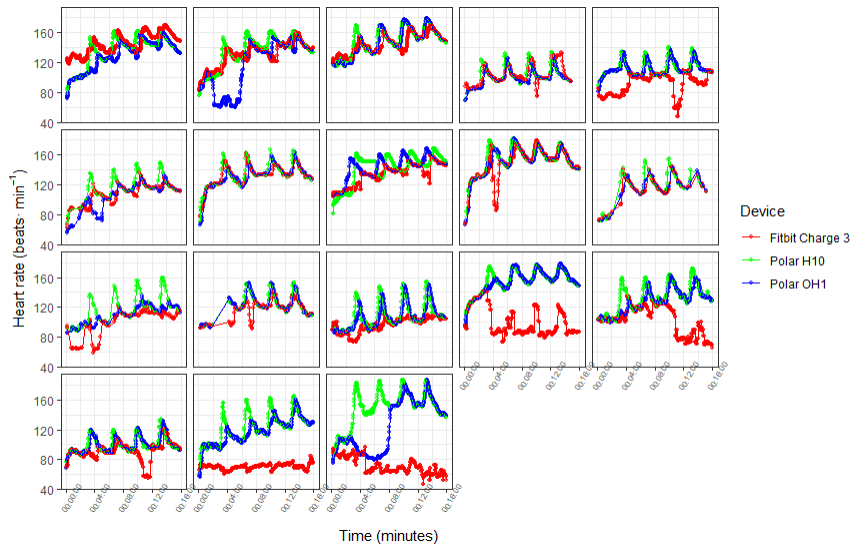
Individual traces during sprint cycling. Green line represents Polar H10 (criterion device), the blue line represents Polar OH1, and the red line represents Fitbit Charge 3. Traces show 4 peaks in heart rate for each of the sprints followed by a recovery period of 3 minutes.

The mean bias and LoA for Polar OH1 and the criterion device were consistent for visit 1 activities; however, the LoA was much wider during HIIE exercise (Figure 1). Similarly, Polar OH1 performed consistently across each segment of visit 1, with very low MAPE and a very good correlation (Table 1). The MAPE and correlation coefficients were worse during HIIE activities (Table 1; Figure 1). Fitbit Charge 3 performed well with a low MAPE (<5%) and had a good correlation during the rest and treadmill activities. Although the MAPE was low for sprint cycling and sprint running, the correlation was poor (Table 1). Validity was poor during light cycle exercise (*r*=0.272; MAPE=−7.1%) and was very poor during vigorous cycling (*r*=0.183; MAPE=−16.4%). The mean bias ranged from −21 to −1 beats·min^-1^ and, together with the LoA, are depicted in Figure 1. Validity across participants was consistent for OH1, where the MAPE was <5% for each participant (Table 3). The MAPE for each participant for Fitbit Charge 3 exceeded 5% in 7 of 19 participants (Table 3). Individual traces for each of the 3 devices during sprint cycling are shown in Figure 2.

**Table 3:** The mean absolute percentage error for each participant for Polar OH1 and Fitbit Charge 3 across all available data points.

Participant	Polar OH1 (%)	Fitbit Charge 3 (%)
1	0.22	6.34^a^
2	−3.92	5.30^a^
3	1.37	2.80
4	0.06	2.06
5	0.31	4.01
6	1.70	1.53
7	0.22	2.35
8	−0.44	2.59
9	0.71	3.14
10	−0.09	2.83
11	0.59	-3.25
12	1.81	4.00
13	−0.10	3.91
14	−0.09	−5.43^a^
15	0.69	14.90^a^
16	0.87	5.97^a^
17	0.19	2.75
18	1.18	11.40^a^
19	2.58	15.80^a^
Overall	0.41	4.37

^a^Exceeds 5% mean absolute percentage error threshold.

## Discussion

### Principal Findings

To our knowledge, this is the first study to assess the validity of Fitbit Charge 3 and Polar OH1 across a range of activity types, including HIIE or SIE. The main findings were that Polar OH1 showed good agreement in assessing HR versus the criterion measure (Polar H10) across activity domains or types in trial 1, whereas the validity of Fitbit Charge 3 was only acceptable during rest and treadmill activities. Fitbit Charge 3 performed particularly poorly during cycling exercise, where the mean bias ranged from −7 to −21 beats·min^-1^, and LoA were very wide compared with other activity types. Finally, our data suggest that both Polar OH1 and Fitbit Charge 3 devices performed poorly during the visit 2 sprint cycling and sprint running protocols compared with the visit 1 activities.

The findings in this study suggest that Polar OH1 performs within acceptable tolerance limits for measuring HR during a range of activity types and intensities (ie, MAPE range 0%-4%). These findings are consistent with those of previous work [7]. Despite this, our data suggest that OH1 may be less capable of accurately measuring HR during sprint-based exercise.

Figure 2 shows the individual responses to sprint cycling for each of the 3 devices. Polar OH1 followed a similar trend to Polar H10 in 13 of the 18 available data sets. In contrast, Fitbit Charge 3 only followed a similar profile to Polar H10 in 5 of the 18 participant data sets. A typical observation across participants was a lower peak HR versus the criterion device and a slower response in HR change following the onset of sprint work. Given the popularity of HIIE and SIE training, these limitations may be crucial for assessing intensity accurately, and further development of the technology is required before it can be used accurately instead of ECG-type devices across all activity types. For everyday activities and for measuring HR in a range of activities, PPG-type devices may therefore be a more practical solution for estimating the intensity of activities. This may be particularly relevant in older, overweight or obese, or clinical populations, where wearing a chest strap may be off-putting or impractical.

This is the first published study to examine the validity of Fitbit Charge 3, although several studies have investigated the validity of its predecessors, Fitbit Charge HR (released 2015) and Fitbit Charge 2 (released 2016). We observed HR measurements that were within an acceptable percentage error range (0%-5%) during rest and the incremental treadmill test. However, Fitbit Charge 3 exhibited MAPE >5% during sprint running and during light, hard, and sprint cycling, with particularly large MAPE and mean bias observed during hard and sprint cycling. Previous studies have reported that Fitbit Charge HR and Fitbit Charge 2 underestimate HR in comparison with criterion devices during cycle-based activities [8,9,11,14]. In addition, increasing exercise intensity appears to increase Fitbit Charge HR and Charge 2 measurement errors [8-10,14,23-25]. Furthermore, Reddy et al [9] reported that Fitbit Charge 2 criterion-related validity was poor during cycling when transitioning swiftly from low to high intensity, which is in agreement with our cycling SIE findings, suggesting that Fitbit Charge model exhibits a measurement lag when HR increases rapidly. Therefore, under the conditions of this study, Fitbit Charge 3 performs as poorly as its predecessors when measuring HR during cycling activities and high-intensity exercise.

We can only speculate as to why Fitbit Charge 3 performed worse than Polar OH1 during cycling and high-intensity exercise in this study. Olstad and Zinner [26] previously suggested that wrist-based devices may be less sensitive to sudden changes in exercise intensity, such as when transitioning from low to high intensity, as experienced in SIE, because peripheral resistance is lower at the wrist, reducing changes in pulse pressure and disrupting blood pulse detection [16]. Therefore, the positioning of Polar OH1 may partially mitigate the poor signal detected at the wrist location. Furthermore, greater movement at the wrist may contribute to poor measurement accuracy [8,9,27], particularly during activities involving sustained hand and forearm muscle contractions [15], as may be experienced when gripping the handlebars during intense cycling. It is possible that Polar OH1 is less prone to movement artifacts because of a more robust strap design, which Spierer et al [15] previously identified as a potential contributor to differences in HR measurements among devices. In addition, Thomson et al [10] stated that hardware differences may affect the signal-to-noise ratio of the device. PurePulse technology of Fitbit Charge 3 may be more sensitive to interference than the technology of Polar OH1, which could have resulted in the less accurate HR measurements obtained in this study. Further advancements in technology are inevitable and will require further work from the scientific community to scrutinize devices and software advancements. Nevertheless, PPG-based devices provide an exciting opportunity to improve physical activity levels, promote adherence to exercise interventions, and drive behavior change.

This study included a variety of exercise intensities and both cycling and treadmill activities. In addition, few studies have examined the validity of consumer wearables in measuring HR during SIE, an increasingly prevalent exercise modality. However, despite providing novel insights into the accuracy of Fitbit Charge 3 and Polar OH1 in the detection of HR, this study has limitations. The sample size of this study, which is consistent with other similar studies [6,7,13,26,28-30], is small; therefore, the elements of the study are likely underpowered. This is particularly true for the HIIE type exercise, where a large variation in HR is observed, which is far from zero. As a result, it is inevitable that the associated LoAs are large and that a larger sample size is required to reduce the variation in the measurement. We refer readers to a new consensus statement by Mühlen et al [31] for further reading regarding sample size estimation for validation studies and for the design of validation studies in general. Furthermore, given the significant effort and time required to perform these types of studies, adequate funding is required from manufacturers to appropriately address the issue of sample size in validation studies. Other limitations include that the exercise tasks were conducted in a controlled laboratory environment, and the performance of these devices may differ when investigated under free-living conditions. This study, similar to many activity tracker validation studies, elected to recruit a cohort of individuals who were healthy and young to middle aged (25-56 years). Therefore, our findings cannot be generalized to clinical populations or different age groups. Furthermore, although not always the case [32], skin tone has been found to affect PPG signals [33] but was not accounted for in this study. Finally, our study design did not assess device acceptability, which should be considered if these devices were to be used in community-based interventions.

### Conclusions

In conclusion, our data suggest that Polar OH1 is a suitable method for measuring HR during cycling, walking, and running activities within a healthy population. In contrast, data pertaining to Fitbit Charge 3 should be interpreted with caution, particularly during cycling activities. This may have significant implications for exercise training or rehabilitation purposes, where attainment of exercise intensity is a key aspect for cardiorespiratory fitness progression or where safety considerations exist. Furthermore, both PPG sensors evaluated in this study performed worse during the SIE activities. Given the rise in popularity of HIIE or SIE, we recommend that more traditional ECG/HR monitors are used when performing these activities. For the general population and scientific community to appropriately interpret PPG data, researchers should continue to assess the validity of new and existing devices among various populations and settings.

## Appendix

Multimedia Appendix 1 Wattbike resistance settings.

Multimedia Appendix 2 Sprint running distance settings.

Multimedia Appendix 3 Sprint running treadmill belt resistance (parachute size) settings.

## Abbreviations

ANS: autonomic nervous system
ECG: electrocardiogram
HIIE: high-intensity interval exercise
HR: heart rate
IPAQ-SF: International Physical Activity Questionnaire-Short Form
LoA: limits of agreement
MAPE: mean absolute percentage error
PPG: photoplethysmography
SIE: sprint interval exercise
