# Supplementary figures and images for: Measurement of Heart Rate Using the Polar OH1 and Fitbit Charge 3 Wearable Devices in Healthy Adults During Light, Moderate, Vigorous, and Sprint-Based Exercise: Validation Study
Source: JMIR Mhealth Uhealth. 2021 Mar 25;9(3):e25313. doi: 10.2196/25313 (PMC8088863; doi:10.2196/25313)

**Multimedia Appendix 1.** Wattbike resistance settings.

**
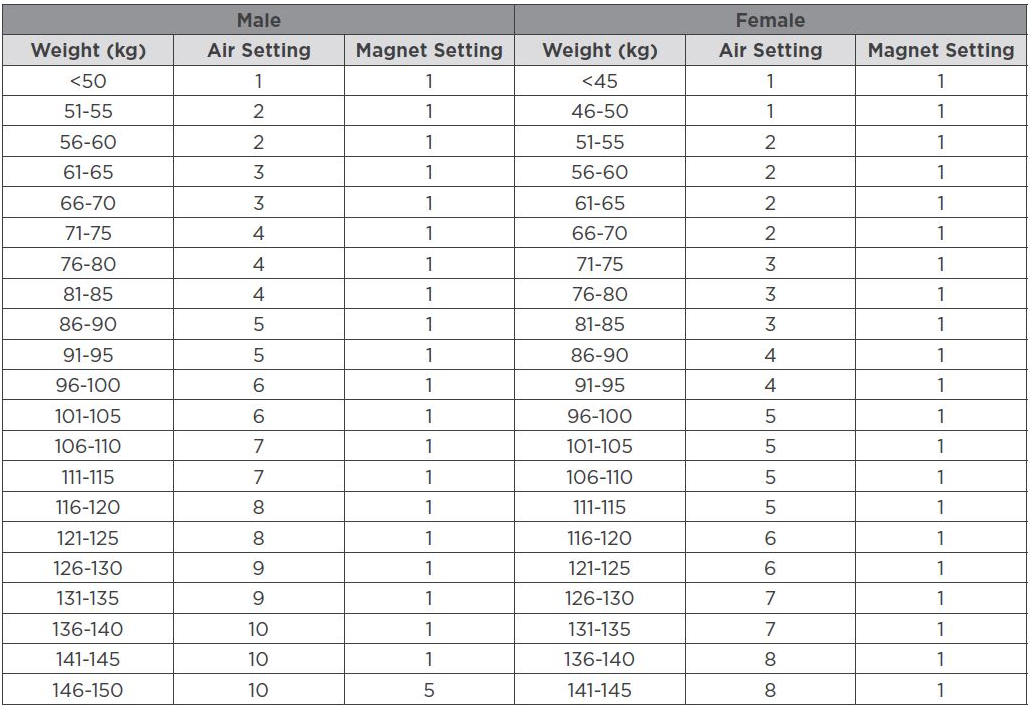
**

Supplement: Multimedia Appendix 1 [file mhealth_v9i3e25313_app1.docx]
