# Supplementary material for: Measurement of Heart Rate Using the Polar OH1 and Fitbit Charge 3 Wearable Devices in Healthy Adults During Light, Moderate, Vigorous, and Sprint-Based Exercise: Validation Study
Source: JMIR Mhealth Uhealth. 2021 Mar 25;9(3):e25313. doi: 10.2196/25313 (PMC8088863; doi:10.2196/25313)
Supplement: Multimedia Appendix 2 [file mhealth_v9i3e25313_app2.docx]

**Multimedia Appendix 2.** Sprint running distance settings.

| **Visit 1 Treadmill Speed (km/h)** | **Distance (m)** |
| --- | --- |
| 10.0 | 30 |
| 12.0 | 35 |
| 14.0 | 40 |
| 16.0 | 45 |
| 18.0 | 50 |
