# Supplementary material for: Measurement of Heart Rate Using the Polar OH1 and Fitbit Charge 3 Wearable Devices in Healthy Adults During Light, Moderate, Vigorous, and Sprint-Based Exercise: Validation Study
Source: JMIR Mhealth Uhealth. 2021 Mar 25;9(3):e25313. doi: 10.2196/25313 (PMC8088863; doi:10.2196/25313)
Supplement: Multimedia Appendix 3 [file mhealth_v9i3e25313_app3.docx]

**Multimedia Appendix 3.** Sprint running treadmill belt resistance (parachute size) settings.

| **Body mass (kg)** | **Males** | **Females** |
| --- | --- | --- |
| <61 | Small | X-Small |
| 61-80 | Medium | Small |
| 81-100 | Large | Medium |
| >100 | X-Large | Large |
